# Using Cocreation in the Process of Designing a Smartphone App for Adolescents and Young Adults With Cancer: Prototype Development Study

**DOI:** 10.2196/formative.9842

**Published:** 2018-11-01

**Authors:** Abbey Elsbernd, Maiken Hjerming, Camilla Visler, Lisa Lyngsie Hjalgrim, Carsten Utoft Niemann, Kirsten Arntz Boisen, Jens Jakobsen, Helle Pappot

**Affiliations:** 1 Department of Oncology Rigshospitalet Copenhagen University Hospital Copenhagen Denmark; 2 University of Kansas School of Medicine Kansas City, KS United States; 3 Department of Hematology Rigshospitalet Copenhagen University Hospital Copenhagen Denmark; 4 Department of Pediatric Hematology and Oncology Department of Pediatric and Adolescent Medicine Rigshospitalet, Copenhagen University Hospital Copenhagen Denmark; 5 Center of Adolescent Medicine Department of Pediatric and Adolescent Medicine Rigshospitalet, Copenhagen University Hospital Copenhagen Denmark; 6 Daman Copenhagen Denmark

**Keywords:** adolescent and young adult, cancer, cocreation, mHealth, oncology

## Abstract

**Background:**

Adolescent and young adult (AYA) oncology and hematology is a developing field of medicine, focusing on a population that faces many challenges throughout medical treatment and beyond. Mobile health (mHealth) interventions provide exciting new opportunities for improvement of health-related quality of life (HRQoL) in AYAs with cancer. Many smartphone apps are currently available for AYAs with cancer; however, for AYAs with cancer, very few apps have been designed with direct input from AYAs themselves or have demonstrated their effectiveness and benefit.

**Objective:**

The objective of this project was to develop the prototype of a smartphone app for AYAs with cancer through the process of cocreation, with the active input of AYAs who have received treatment for cancer directly impacting content and design.

**Methods:**

Patients were recruited from a population of Danish AYAs who had received treatment for cancer between the ages of 15 and 29 years. The cocreation process was completed over the course of 3 workshops and intermittent ad hoc meetings, where the recruited AYAs worked in coordination with 1 nurse, 1 doctor, and 2 representatives from a digital agency and app developer. During each workshop, participants prioritized their goals for the app. After new app content was developed, feedback was requested from the participants, and changes were made accordingly. This iterative process continued until consensus on final product features and design were achieved. Health care professionals provided minimal input and primarily performed observational roles in the workshops, with direct interaction limited to introducing the project and explaining measurement features of the app in development.

**Results:**

Three key features to be included in the prototype app were identified from the cocreation workshops: (1) a community forum; (2) an information library; and (3) a symptom and side-effect tracking tool. Bright, warm colors were selected for the app by the participating AYAs. The final prototype will be launched for pilot testing and implementation testing in February of 2018.

**Conclusions:**

The process of cocreation is a user-involved process that can create an end product that is useful and customized for the target population. This process, as such, is a beneficial process to utilize when addressing the specific needs of AYAs with cancer. The results of the here described app prototype will be evaluated in more detail in the near future. However, this description of the cocreation process in app development can be utilized for the creation of other mHealth interventions.

## Introduction

Adolescent and young adult (AYA) oncology and hematology is a developing field of medicine, focusing on a population that faces many distinct hardships throughout medical treatment and beyond. AYAs with cancer face many challenges, including those under physical, psychological, and social domains. A cancer diagnosis at any point is devastating, but as an adolescent or young adult, serious disease interrupts a critical period of physical and personal development where relationships, academic, and professional careers, and planning for the future have a significant level of importance [1,2]. As consequence, AYAs with cancer and cancer survivors report lowered health-related quality of life (HRQoL) in comparison to the general population [3-7]. Prior studies have found that AYA transplant patients have equivalent or better HRQoL in comparison to older transplant patients [3], yet both populations experience a drop in HRQoL.

AYA patients are a technologically savvy cohort that feels comfortable communicating and managing their problems and information needs in the digital world [8]. Current advances in technology provide exciting new methods of improving the lives of AYAs with cancer through webpages, smartphone apps, and electronic devices. These resources are designed to provide assistance in symptom tracking, health promotion, and social networking with other patients [9,10]. One of the more ubiquitous technology interventions used in the AYA cohort is that of the smartphone app, under the umbrella of mobile health [11]. Apps are a useful platform for AYAs due to portable access and the rise of smartphone utilization in the AYA age group [12-15]. Many apps are currently available for AYAs with cancer [16]; however, the effect of this health intervention approach is understudied. Few out of the hundreds of available smartphone apps for AYAs with cancer have demonstrated their effectiveness and benefit in the currently available literature, and few have been designed with direct input from AYAs themselves in a complete and thorough fashion [15-19]. Even with existing resources, there is still room and availability to expand patient technology options [11].

In order to create a smartphone app that would be useful and engaging, the input and involvement of end users in the app’s development is imperative [20,21]. Kræftværket, named after the Danish words for “power plant” (Kraftværk) and “cancer” (Kræft), is a youth-friendly sanctuary for AYAs with cancer aged 15-29 years at Rigshospitalet in Copenhagen, Denmark. It was designed from a cocreation-based “hackathon” event where designers and AYAs with cancer or prior cancer experience worked together to design youth-oriented facilities. This process allowed AYAs to play an active role in the creation of their own environment, thus empowering them throughout the time of disease [22,23]. It is this mindset—providing young patients with cancer with the tools to create their own preferred intervention—that inspired the research team to develop an mobile health intervention via the process of cocreation. Cocreation gives key decision-making capacity on app design and content to the target audience of the end product. This allows the young people who intend to utilize the app to become active contributors in their own desired outcome, bringing forward unique ideas and experiences that the health care team may not have [24].

The aim of this project was to develop a prototype of a smartphone app for AYAs with cancer through the process of cocreation; we here describe the cocreation process and how this procedure was used to develop specific youth-oriented features and directly involve AYAs. The resulting app prototype is entitled “Kræftværket,” after its namesake youth sanctuary.

## Methods

### Recruitment

At all phases of the cocreation process, we continuously recruited patients from a population of Kræftværket users, consisting of Danish AYAs with cancer and cancer survivors aged 15-29 at the time of diagnosis who have received treatment at Rigshospitalet. Patients aged >29 years were eligible if they were diagnosed with cancer between the ages of 15 and 29 years. Patients currently receiving treatment and those no longer receiving treatment were eligible. An open invitation to participate in the cocreation workshops was posted by a hospital youth coordinator via the closed Kræftværket Facebook group before each event for recruitment. This method resulted in a combination of prior participants and new participants during the cocreation workshops. Patients were excluded from participation if they were unable to read or communicate in Danish.

### The Cocreation Process

The app was developed via a cocreation process, in which young people defined the goals of a technology intervention and then had direct involvement on design and key features. Cocreation is a design principle in which the target consumer of a product or resource plays a principle role in an end product’s formation [25]. This process expands on the ideology introduced in principles of user-centered design; however, in user-centered design, the target users typically play a passive role while being interviewed or observed by area experts [26]. Cocreation instead places the driving force with the target user.

**Figure 1:**
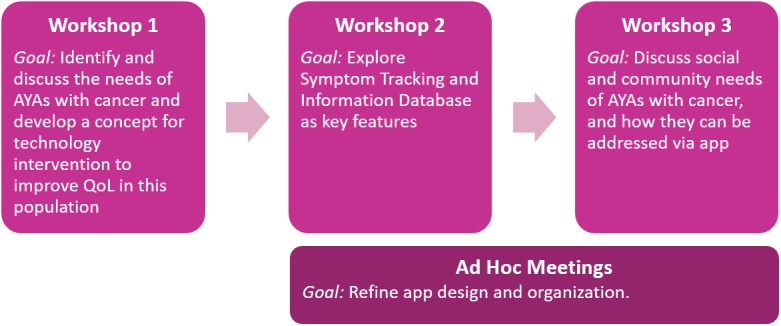
Flow chart of cocreation meetings. AYA: adolescent and young adult; QoL: quality of life.

**Figure 2:**
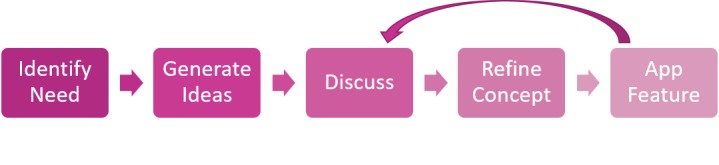
Flowchart detailing cocreation process utilized in workshop series.

The cocreation process was completed over the course of 3 workshops and intermittent ad hoc meetings, where the recruited youth panel worked together with a nurse, a doctor, and 2 representatives from the digital agency and app developer Daman. Workshops were selected as the primary cocreation method due to prior experience from the app developer and research team. The health care professionals did not actively participate in the workshops and only observed the events. Observers took notes on their observations of key issues discussed and relevant to AYAs with cancer. Representatives from the digital agency played a facilitative role, offering questions and guiding discussion based on the goals for the current workshop. Goals of the 3 workshops are outlined in Figure 1. Workshops were held in nonhospital environments (eg, cafés and restaurants), while ad hoc meetings were held at the Kræftværket day room facilities at Rigshospitalet.

Before each workshop, participants were informed of the end goals of this project and specific goals of individual workshops. Specific content of each workshop was dynamic throughout this process and determined based upon the status of the app in development, as well as goals prioritized by youth during the current workshop or at workshops prior. No materials were needed for preparation of workshops excluding food and beverages for participants. Further detail on the content of each workshop is described in the results.

The cocreation process involved AYA participants at all levels of the project. During each workshop, participants described the needs of AYAs with cancer. The group was then asked to present ideas involving how these needs can be addressed using an app intervention. The group then discussed these ideas, refining the concepts and prioritizing ideas deemed most beneficial by the group. The culmination of these discussions would then be integrated into an app feature or design aspect. This ensured a functional tool that is both usable and meaningful for the target users. When a design or functional change of any feature was made, the results were presented to participants to reassess, discuss, and then approve or disapprove of the current feature or design choice. Figure 2 outlines the general structure of the cocreation process.

After each workshop and ad hoc meeting, collaborative efforts were made between the digital agency and the research team to create a finished app prototype reflecting the ideas and discussions of participating AYAs. The final app is expected after an evaluation phase or pilot test, as feedback throughout the next steps of the development process will be used to modify the current app prototype.

### Ethical Considerations

All participants have completed informed consent forms prior to participation in any study procedure. If a participant was aged <18 years, caregiver informed consent was additionally obtained. This research was exempt from review by an institutional review_board_ or ethical authority under Danish law. All procedures performed in studies involving human participants were in accordance with the ethical standards of the institutional and national research committee and with the 1964 Helsinki declaration and its later amendments or comparable ethical standards.

## Results

### Demographic Characteristics

Over the course of 3 workshops, 17 individual AYAs participated. The participants had an average age of 23.9 (range 17-32) years. Among the participants, 10 identified as male and 7 identified as female. Moreover, 9 participants had a hematologic diagnosis, while 8 had an oncologic diagnosis. Diagnoses varied and included leukemia, lymphoma, breast cancer, testicular cancer, primary brain cancer, adrenocortical cancer, and neuroendocrine cancer. Each workshop was composed of participants who were on and off treatment. Due to the continuous recruitment process, 10 participants attended 1 workshop, 4 attended 2 workshops, and 3 attended 3 workshops. Complete demographics per event are listed in Table 1. Ad hoc meetings were open and held briefly at the hospital; 2-3 AYAs participated in each of the 3 ad hoc meetings.

### Workshop I

Workshop I was performed over the course of a weekend retreat, with approximately 10 hours dedicated to cocreation. During the initial workshop, the AYA panel members were asked to describe an ideal technology resource that could be used to improve quality of life for AYAs with cancer and what resources would be most beneficial outside of the Kræftværket social room and closed Facebook group. During the workshop, participants identified needs that an electronic health intervention could address by answering questions such as the following: What is at stake when diagnosed with cancer as a young person? What is everyday life like with cancer? What is it like to undergo a course of treatment? Lastly, what digital tool could address the needs of a young person with cancer? A mobile app was confirmed as the most beneficial technology platform.

### Workshop II

Workshop II was held during an afternoon meeting, with approximately 5 hours dedicated to cocreation. In this workshop, AYAs defined their primary information needs during cancer treatment and what validated knowledge they believed should be accessible via the app. In addition, they were asked to identify what logging features, such as “”Pain” or “Mood,” should be available in the symptom tracking feature of the app. Participants were divided into 2 discussion groups to brainstorm ideas, and the generated ideas were then narrowed and ranked in terms of importance. For both the symptom tracking parameters and the information resources, participants were asked to select the top 10 most important brainstormed ideas and then rank them in the order of most to least important. The 10 information resources and tracking parameters deemed most important overall were then integrated into the pilot app product.

### Ad Hoc Meetings

**Figure 3:**
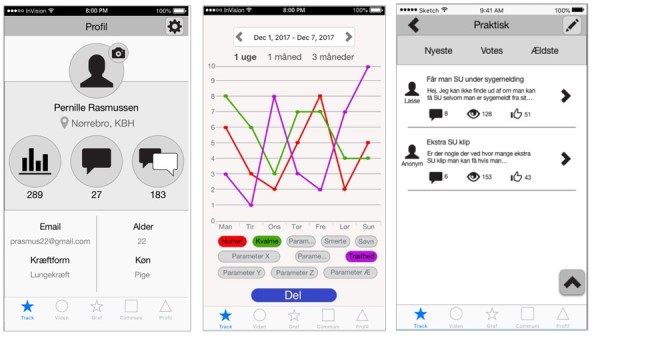
Wireframe of the initial design for the app prototype used during cocreation ad hoc meetings.

During the time period between the second and third meeting, ad hoc meetings were arranged at Kræftværket over lunch to discuss the app’s design. During these meetings, Kræftværket users were approached to clarify, test, and evaluate different graphic designs available. They were also asked to assess development wireframes, which are blueprints of an app’s visual content and navigation elements (Figure 3). Feedback gathered from ad hoc meetings allowed the app development team to quickly adjust the design and functionality and gain direct feedback from anticipated app users.

### Workshop III

Lastly, Workshop III was held at an afternoon meeting, with approximately 5 hours dedicated to cocreation. At the final cocreation workshop, the aim was to identify how AYAs with cancer communicate with one another and how this can be utilized on a digital platform to share experiences and provide advice. Smaller discussion groups were formed to perform specific activities designed to highlight desirable features for a community-based app. Activities included “Brainwriting,” in which patients were asked to write down as many thoughts as they could on communication between AYAs with cancer in a 10-minute span, as well as “Dotting,” where patients narrowed the generated ideas by selecting those that they deemed most personally significant. The social and community features were refined for the app after the workshop’s completion.

**Table 1:** Participant demographics throughout cocreation workshops.

Characteristics			Workshop 1 (n=12)	Workshop 2 (n=9)		Workshop 3 (n=6)
Age (years), mean (SD)			24.44 (4.6)	23.11 (4.5)		23.17 (3.2)
**Gender, n (%)**						
	Female	5 (42)		3 (33)	1 (17)	
	Male	7 (58)		6 (67)	5 (83)	
**Diagnosis, n (%)**						
	Hematologic	7 (58)		5 (56)	3 (50)	
	Oncologic	5 (42)		4 (44)	3 (50)	
**Treatment status, n (%)**						
	On treatment	9 (75)		4 (44)	2 (33)	
	Off treatment	3 (25)		5 (56)	4 (67)	

**Information library items identified by participants from cocreation workshop and integrated into the app prototype.**

- What is cancer?
- Medication and treatment
- How to disclose and discuss cancer diagnoses with family and friends?
- Hospitalization
- Navigating economic, municipal, and educational systems
- Alternative medicine
- Health and nutrition
- Hobbies and activities
- Cosmetics and personal grooming

In the final pilot prototype, 3 key app features were identified from the cocreation workshops: (1) a community forum; (2) an information library; and (3) a symptom and side-effect tracking tool. Design-wise, the participants selected a bright, warm color scheme reminiscent of the physical Kræftværket facilities that were initially chosen during Kræftværket’s hackathon creation event [22].

The community forum is intended to serve as an open community network where AYAs with cancer can connect with peers. Participants stated that they would prefer a private area to speak with others who understand their situation, while also providing the freedom allotted in the form of an open forum. Private messaging features will also be included. Cocreation participants at the third workshop discussed the creation of a mentoring feature in which new users can be matched with someone of similar diagnosis to privately connect with. However, this feature was not included in the final app prototype due to financial, technical, and ethical limitations.

App users identified 10 items deemed important to include in an information library, as outlined in Textbox 1. Links to outside resources were also suggested. Participants asked for informative links to hospital and governmental websites, as well as the ability for the app to suggest nearby activities, restaurants, and locations to visit when hospitalized.

Symptom and side-effect tracking were seen as important ways to monitor personal well-being as well as to provide a tool to explain side effects and symptoms in visits at the hospital with health professionals. Participants identified 5 initial trackable features, including sleep, pain, fatigue, nausea, and mood. Participants suggested customization of what symptoms and side effects could be tracked for a more personal experience to each individual that can be followed over time. As a consequence, the symptom tracking can have different metrics added and removed to the desire of the app user.

By this process, the Kræftværket app was completed for utilization both during and after cancer treatment on both iOS and Android platforms. The final prototype has been moved to pilot testing and evaluation, with results expected in future publications [27].

## Discussion

### Principal Findings

Based on the described cocreation process, a prototype for the Kræftværket smartphone app was developed. It is intended to serve as a tool for AYAs with cancer to improve quality of life during and after cancer treatment, as well as form a supportive community with other AYAs.

As designated by Bandura’s theory of self-efficacy, perceived self-efficacy is defined as people’s beliefs about their capability to influence change in their lives [28]. Cocreation addresses this by giving patients back the authority to address personal challenges via direct involvement in supportive resource creation, thus benefiting self-efficacy for both those involved in the process and the end users [29-32]. The utilization of cocreation, in addition, does more than simply provide an avenue for patient self-efficacy and agency. Increased participant input also allows for the creation of an end product that is useful, patient-centered, and engaging [15,19,21,33].

**Figure 4:**
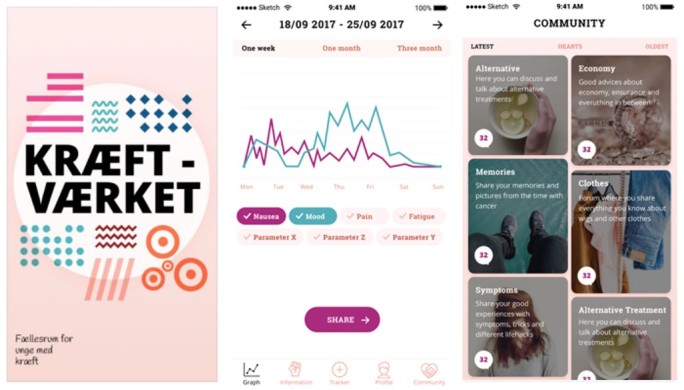
Screenshots of current development model for the Kræftværket smartphone app.

Currently the app prototype is under evaluation via ongoing pilot testing and an implementation test evaluating quality of life via the European Organization for the Research and Treatment of Cancer Quality of Life Questionnaire-Core 30 (Figure 4) [27,34]. The hypothesis for the Kræftværket app is that in the long term, the app can serve as a patient support tool and assist in meeting many patient needs, including side-effect and late-effect management, handling existential and practical concerns, and confronting daily life with cancer in a supportive community of peers. This is proposed to benefit the global health status and functional scale domains of the European Organization for the Research and Treatment of Cancer Quality of Life Questionnaire-Core 30 while also improving the management of symptoms through tracking to a lesser extent under the symptom scales domain [34]. These social, psychological, and functional concerns have been identified as areas of concern for AYA patients that may be addressed by intervention [35,36]. However, this has yet to be investigated from the Kræftværket app. We hope that participants utilizing the Kræftværket app will report higher HRQoL using a standardized measurement in comparison to control groups and will qualitatively report that the Kræftværket app has benefited them in psychosocial and practical domains.

The cocreation approach is an approach to create user-friendly interventions, providing strength in development of a product that is both functional and desirable to its target audiences. For smartphone app interventions, a functional product does not necessarily guarantee success or frequent app usage [17]. Based on feedback from AYAs during the cocreation workshops, decisions may change, and the youth may orient the project in ways that the health care professionals and representatives from the digital agency may not have chosen on their own. As such, the project may be seen as more desirable and functional, addressing the needs from a user-focused perspective.

### Strengths and Limitations

The cocreation process is one of the greatest strengths of this project; however, cocreation may also serve as a limitation. By giving significant decision-making to the youth, the ability to follow a rigid protocol of development is somewhat hindered; throughout this project, AYAs played an equally significant role in creating the prototype as any professional or area expert. However, we believe that the benefits of cocreation outweigh the limitations. In further studies, by adding quantitative surveys examining HRQoL, there will be a tangible method to analyze the impact of the project’s end results and therefore the utility of the cocreation process.

The continuous recruitment process was an additional limitation, as the population was constantly fluctuating. A decrease in participant number was noted with each subsequent cocreation workshop, which may be attributable to the decreased time for recruitment with each subsequent workshop, and participant availability, as the initial workshop was held over a weekend while each subsequent workshop was on a weekday afternoon into the evening. Additionally, a different composition of AYAs was noted in each workshop, which may also be a limitation due to the inability for one group to comment on refinements to the app concept. Lastly, small sample sizes were provided for cocreation workshops, which may not be adequately heterogenous to represent all AYAs with cancer. However, the quantitative and qualitative analyses that will be added in the prototype’s evaluation will provide an opportunity to extend this project to a larger population of AYAs, who will be targeted during recruitment to achieve a more representative population of all AYAs with cancer.

It is the aim of the authors of this article to describe the development of an app for AYAs with cancer or cancer experience; however, the described cocreation process will be applicable for the development of applications and other interventions for AYAs with other chronic diseases. Previous studies have showed improved target outcomes via app utilization for disease management, such as blood glucose levels in diabetes and improved asthma control [37]. The exact role that smartphone apps may have on AYAs’ HRQoL is not quite clear. In addition, not all health applications are created in an equal process; many applications are not validated through evidence-based testing, and those that have been evaluated do not always report improvement of target health outcomes [37,38]. However, the potential remains for smartphone apps to serve as a new strategy for improving HRQoL and other health outcomes for broader populations of AYAs beyond those with cancer. While it is true that there are many applications currently available for AYAs with chronic diseases, there are few that have utilized AYA input in their development [18]. By applying the described process to a wider range of diagnoses and chronic illnesses, there is potential to develop a cocreated application with significant benefit to young people regardless of diagnosis.

### Conclusions

In conclusion, using the process of cocreation, the prototype smartphone app Kræftværket was designed as an integrated tool for AYAs with cancer and cancer survivors. Further research and analysis are ongoing to evaluate the effect of this application on HRQoL. The application and design process have potential to serve as inspiration for the development of other interventions with a user-involved method; however, more evaluation is needed.

## Abbreviations

AYA: adolescent and young adult
HRQoL: health-related quality of life
mHealth: mobile health
